# In-vitro antiviral activity of *Solanum nigrum *against Hepatitis C Virus

**DOI:** 10.1186/1743-422X-8-26

**Published:** 2011-01-19

**Authors:** Tariq Javed, Usman Ali Ashfaq, Sana Riaz, Sidra Rehman, Sheikh Riazuddin

**Affiliations:** 1 Division of Molecular Medicine, National Centre of Excellence in Molecular Biology, University of the Punjab, Lahore, Pakistan; 2 Department of Chemistry, Government College University, Lahore, Pakistan; 3 Allama Iqbal Medical College, Allama Shabir Ahmad Usmani Road, Lahore, Pakistan

## Abstract

**Background:**

Hepatitis C is a major health problem causes liver cirrhosis, hepatocellular carcinoma and death. The current treatment of standard interferon in combination with ribavirin, has limited benefits due to emergence of resistant mutations during long-term treatment, adverse side effects and high cost. Hence, there is a need for the development of more effective, less toxic antiviral agents.

**Results:**

The present study was designed to search anti-HCV plants from different areas of Pakistan. Ten medicinal plants were collected and tested for anti-HCV activity by infecting the liver cells with HCV 3a innoculum. Methanol and chloroform extracts of *Solanum nigrum *(SN) seeds exhibited 37% and more than 50% inhibition of HCV respectively at non toxic concentration. Moreover, antiviral effect of SN seeds extract was also analyzed against HCV NS3 protease by transfecting HCV NS3 protease plasmid into liver cells. The results demonstrated that chloroform extract of SN decreased the expression or function of HCV NS3 protease in a dose- dependent manner and GAPDH remained constant.

**Conclusion:**

These results suggest that SN extract contains potential antiviral agents against HCV and combination of SN extract with interferon will be better option to treat chronic HCV.

## Introduction

An estimated 3% of the world's population (270 million people) is chronically infected by HCV which is the main cause of liver fibrosis and cirrhosis that leads to hepatocellular carcinoma (HCC) in a significant number of patients [1,2]. Almost 10 million people in Pakistan are living with HCV [3] and the most prevalent HCV genotype is 3a followed by 3b and 1a [4]. HCV is enveloped, positive strand RNA genome comprising 9.6 kb of uncapped RNA [5,6]. The internal ribosomal entry site (IRES) is located within the 5'UTR of the HCV genome that initiates translation of a large precursor polyprotein, which is processed by cellular and viral proteinases to form 10 viral proteins, specifically Core, E1, E2, p7 (structural proteins), NS2, NS3, NS4a, NS4b, NS5a and NS5b (nonstructural proteins) [6-8]. The nonstructural proteins (NS2, NS3, NS4A, NS4B, NS5A, and NS5B) provide enzymes essential for protein processing and RNA replication; their functions include protease, RNA helicase, and RNA polymerase activity [9].

However, there is no vaccine available for HCV and 40-50% of patients fail to respond to current therapies of PEG-INF/Ribavirin [10]. Neither interferon (INF) monotherapy, nor a combination of IFN or ribavirin, have been able to eradicate HCV replication in the majority of patients [11]. The modified forms of IFN, such as Pegylated IFN, etc. are available and the rate of sustained virologic response (SVR) in the patients receiving Pegylated-interferon α was 39% [12]. The SVR rate for 1a genotype is (about 40-50%) [13] and genotype 2 and 3 is (about 70-80%) [14]. Furthermore, the incidence of adverse effects (including headache, fatigue, myalgia, depression, neutropenia, and thrombocytopenia) in patients receiving PEG interferon was similar to that in patients receiving standard interferon and leads to discontinuation of therapy.

Herbal medicines have been used for centuries against different ailments including viral diseases and become a focal point to identify, isolate and purify new entities to treat diseases like Hepatitis C. According to an estimate, 25% of the commonly used medicines contain compounds isolated from plant origin. The basis of some modern medications is indeed plants, such as aspirin from white Willow bark, digitalis from foxglove, warfarin (Coumadin) from sweet clover, antimalarial drug quinine from the bark of *Cinchona officinalis*, taxol isolated from the Yew plant and digoxin from *Digitalis purpurea *[15]. Medicinal compounds derived from plant extracts, are of lifelong interest to the pharmaceutical industry. For example, taxol is an antineoplastic drug obtained from the bark of the Western yew tree, found to be useful in the treatment of breast cancer [16]. Plants contain a variety of chemically active compounds such as flavonoids, terpenoids, lignans, sulphides, polyphenolics, coumarins, saponins, furyl compounds, alkaloids, polyines, thiophenes, proteins and peptides, which prone to inhibit the replication cycle of various types of DNA or RNA viruses. A survey of presently available and those that are yet to be exploited reveals an innumerable potentially useful phytochemicals waiting to be evaluated and exploited for therapeutic applications against genetically and functionally diverse virus families such as Hepatitis C Virus [17].

The present study is an attempt to lay foundation for screening the potential anti-HCV agents from medicinal plants. For this reason, plant material from ten different traditional medicinal plants were collected, soaked in methanol, concentrated and dried. Different concentrations of extracts lower than 100 μg/μl was checked for toxicity in in-vitro culture of Huh-7 cell line. Antiviral screening of the plant extracts was done on liver cells and HCV RNA (viral load) is determined by Quantitative Real Time RT-PCR. Thus, this information can be useful in the theoretical design of drugs with favorable, improved specificity and activity.

## Materials and methods

### Serum Sample Collection

The local HCV-3a patient's serum samples used in this investigation were obtained from the CAMB (Center for Applied Molecular Biology) diagnostic laboratory, Lahore, Pakistan. Serum samples were stored at -80°C prior to viral inoculation experiments. Quantification and genotype was assessed by CAMB diagnostic laboratory, Lahore, Pakistan. Patient's written consent and approval for this study was obtained from institutional ethics committee.

### Collection and Extraction of Medicinal plants

Ten different plants were selected and dried on the basis of their medicinal characteristics. These indigenous plants were collected from different zones of Pakistan having different habitat. The plant species were legitimated at Department of Botany, University of the Punjab, Lahore. Plants or their parts were dried under shade at room temperature, weighed and macerated in methanol for overnight. Temperature would not exceed from 38°C which is the most desirable temperature for enzymatic activity. After 24 h solvents were filtered, residue was soaked again in fresh solvent. Process of filtration was repeated over 3-4 days. Methanolic extracts exhibiting antiviral activity were further partitioned in Chloroform, Acetone and n-Hexane. Solvents were selected on the basis of polarity for characterization of antiviral compounds. Extracts were weighed and their %age yield was calculated.

### Stock solution preparation

50 mg of each dried plant extract was suspended in one ml of Dimethyl sulfoxide (DMSO) ensuring stock concentration of 50 μg/μl. Sieving the above solution by using 0.22 um filter inside Laminar Flow Hood, storing at -20°C.

### Cell line

The Huh-7 cell line was compassionately offered by Dr. Zafar Nawaz (Biochemistry and Molecular Biology Department, University of Miami, USA). Huh-7 cells were cultured in Dulbecco's modified Eagle medium (DMEM) supplemented with 10% fetal bovine serum & 100 IU/ml penicillin & 100 μg/ml streptomycin, at 37°C in an atmosphere of 5% CO_2_.

### MTT assay for toxicity

To investigate cellular toxicity, 2 × 10^4 ^cells/well was plated into 96-well plates. After 24 h, different concentrations of Herbal extracts were added and the plate was sealed and kept at 37°C in an atmosphere of 5% CO^2 ^for 24 h. After the herbal extracts treatment was over, removed the media and test compounds. 100 μl fresh media and 20 μl of MTT solution (5 mg/ml in PBS) were added to all wells in Columns 1-11. Wrapped the plate in aluminium foil and incubated for 3-4 h at 37°C. Media was carefully removed and added 100 μl of DMSO to dissolve the formazan crystals in Columns 1-11. MTT formazan product was determined by measuring absorbance with an enzyme-linked immunosorbent assay (ELISA) plate reader at a test wavelength of 570 nm and a reference wavelength of 620 nm.

Cell viability was obtained using the following equation:

Percent cell viability=(Test 570nm–620nm/Control 570nm−620nm)*10

### Anti-HCV analysis of plant extracts on Huh-7 cells

Huh-7 cell line was used to establish the in-vitro replication of HCV. A similar protocol was used for viral inoculation as established by Zekari et al. 2009 [18] and El-Awardy et al. 2006 [19]. High viral titer > 1 × 10^8 ^IU/ml from HCV-3a patient's was used as principle inoculum in these experiments. Huh-7 cells were maintained in 6-well culture plates to semi-confluence, washed twice with serum-free medium, then inoculated with 500 μl (5 × 10^7^IU/well) and 500 μl serum free media. Cells were maintained overnight at 37°C in 5% CO_2_. Next day, adherent cells were washed three times with 1 × PBS, complete medium was added and incubation was continued for 48 hrs. Cells were harvested and assessed for viral RNA quantification by Real Time PCR. To analyze the effect of Medicinal plant extracts on HCV infection, serum infected Huh-7 cells were again seeded after three days of infection in 24-well plates in the presence and absence of herbal extracts and grown to 80% confluence with 2 ml medium. After 24 h, cells and total RNA was isolated by using Gentra RNA isolation kit (Gentra System Pennsylvania, USA) according to the manufacturer's instructions. Briefely, cells were lysed with cell lysis solution containing 5 μl internal control (Sacace Biotechnologies Caserta, Italy). RNA pallet was solubilized in 1% DEPC (Diethyl pyrocarbonate treated water). HCV RNA quantifications were determined by Real Time PCR Smart Cycler II system (Cepheid Sunnyvale, USA) using the Sacace HCV quantitative analysis kit (Sacace Biotechnologies Caserta, Italy) according to the manufacturer's instructions.

### Formula for the calculation of HCV RNA concentration

Following formula was used to calculate the concentration HCV RNA of each sample.

$$\frac{\text{Cy3STD}/\text{Res}}{\text{Fam}.\text{ STD}/\text{Res}}\times \text{ coefficient IC}=\text{IU HCV}/\text{mL}$$

IC = internal control, which is specific for each lot.

### Antiviral analysis of SN extract against HCV NS3 Protease

For transfection studies, Huh-7 cells (5 × 10^4^) were plated in 24-well plates for 24 h. The medium was removed and cells were washed with 1× PBS. Cells were transiently transfected with expression plasmids containing HCV NS3 protease in the presence and absence of SN 100 μg extract and interferon by using Lipofectamine™ 2000 (Invitrogen life technologies, Carlsbad, CA) according to the manufacturer's protocol. Total RNA was extracted by using Trizol reagent (Invitrogen life technologies, Carlsbad, CA) according to the manufacturer's protocol. To analyze the effect of SN against HCV NS3 gene, cDNA was synthesized with 1 μg of RNA, using Revert Aid TM First Strand cDNA Synthesis Kit (Fermentas, St. Leon-Rot/Germany). Gene expression analysis was carried out via PCR (Applied Biosystems Inc, USA) by using 2× PCR Mix (Fermentas). Following primers were used for the amplification of HCV NS3 forward primer: GGACGACGATGACAAGGACT; NS3 reverse: CCTCGTGACCAGGTAAAGGT; GAPDH Forward: ACCACAGTCCATGCCATCAC: and GAPDH reverse; TCCACCACCCTGTTGCTGTA PCR was performed by initial denaturation at 95°C for 5 min followed by 30 cycles, each of denaturation at 92°C for 45 s, annealing at 58°C for 45 s, and extension at 72°C for 1 min, with final extension at 72°C for 10 min. The amplified DNA samples were analyzed on 2% agarose gel. The DNA bands were visualized directly under the UV and the photographs of the gels were obtained with gel documentation system.

## Results

Ten medicinal plants were collected from different area of Pakistan on the basis of undocumented reports for antiviral screening against HCV. Plants materials were air dried and extracted in methanol. All information regarding botanical names, family vernacular names, local uses and % yield of ten medicinal plants were shown in Table 1.

**Table 1:** Medicinal plants selected for anti-HCV activity and %age yield

Plants names	Family	Local/Vernacular Names	Local uses	Parts Used	Extracts %yield
*Trachyspermum ammi*	Apiaceae	Ajowan caraway	digestive aid and antiseptic	Seeds	9.7

*Solanum nigrum*	Solanaceae	Black Night Shade, Mako	Treat mouth ulcer, antitumour	Seeds	11.96

*Cichorium intybus*	Compositae	Chakori, kasni	Gallstone, gastro-enteritis, jaundice,	Seeds	9.25

*Phyllanthus amarus*	Euphorbiaceae	Amla	Kidney stone, hypertension, jaundice.	Leaves	7.33

*Schinus molle*	Anacardiaceae	Pink Peppercorns, False pipper nigrum	Antibacterial, antiseptic, diuretic, rheumatism	Fruits	24.65

*Syzygium aromaticum*	Myrtaceae	Clove, lavang	Carminative, anthelmintic, pain killer	Leaves	22.6

*Cordia dichotoma*	Boraginaceae	Clammy cherry, lasoori, gunda,	Anti-inflammatory	Leaves	14.1

*Colocasia esculenta*	Araceae	Kachalu, Arvi	Anti-diarrhea, anorexia, antipyretic.	Leaves	21.5

*Momordica charantia*	Cucurbitaceae	Karela,	Antiviral dyspepsia, constipation,	Leaves	5.9

*Cucumis sativus*	Cucurbitaceae	Kheera, trapush,	Jaundice, mental stress.	Leaves	8.73

### Cellular toxicity through MTT Assay

**Figure 1:**
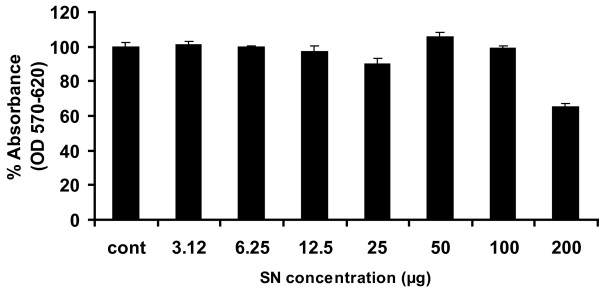
**Toxicity of extract of SN through MTT cell proliferation assay**. Huh-7 cells were plated at the density of 2 × 10^4 ^in 96 well plates. After 24 h cells were treated with different concentrations of herbal extracts and control consisted of solvent in which compound dissolved. After 24 h incubation period add MTT solution to all wells and incubated for 3-4 h at 37°C.Viable cells convert MTT to purple formazan crystal. Added DMSO to dissolve the formazan crystals and read absorbance at 570 nm and 620 nm.

Before starting the antiviral screening against Hepatitis C virus, toxicological effect of ten medicinal plant extracts were determined through MTT cell proliferation assay. The MTT substance is reduced by mitochondrial succinic dehydrogenases in living cells to purple formazan crystals that are not soluble in aqueous water. The absorption of dissolved formazan in the visible region correlates with the number of alive cells [20]. Figure 1 exhibited cytotoxic effects of SN and demonstrated that cell proliferation of liver cells is unaffected up to a concentration of 100 μM. But when we exceeded 100 μM toxic effects were observed. Similar results were observed for additional 9 medicinal extracts ranging from a concentration of 1 to 100 μM.

#### Antiviral Assay

**Figure 2:**
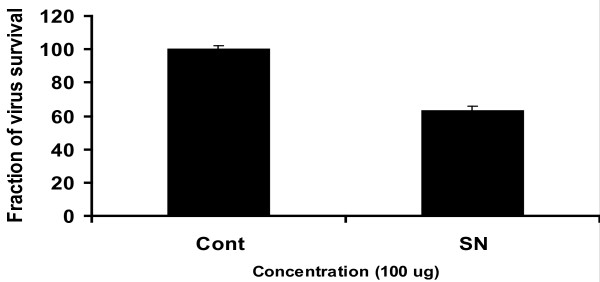
**Anti-HCV activity of methanol extract of *Solanum nigrum***. Huh-7 cells were incubated with HCV serum and 100 μg/μl concentration of *Solanum nigrum *seeds extract for 24 hours. At the end of incubation period, total RNA was extracted by Gentra kit, and the levels of HCV RNA remaining were determined by the Quantitative RT-PCR assay and are shown as a percentage relative to the levels of HCV RNA in cells incubated without compound (control).

**Figure 3:**
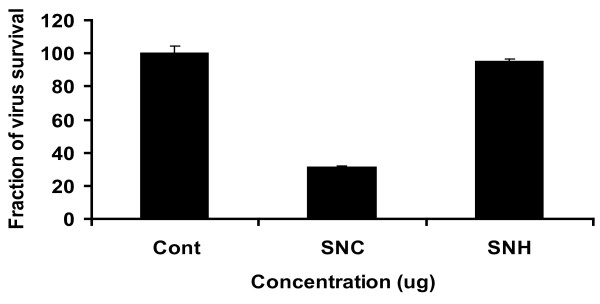
**Anti-HCV activity of chloroform and n-hexane fraction of *Solanum nigrum***. Huh-7 cells were incubated with HCV serum and 100 μg/μl concentration of *Solanum nigrum *seeds extract in different solvents for 24 hours. At the end of incubation period, total RNA was extracted by Gentra kit, and the levels of HCV RNA remaining were determined by the Quantitative RT-PCR assay and are shown as a percentage relative to the levels of HCV RNA in cells incubated without compound (control).

Since HCV replication in cell culture is limited to human hepatocytes and their derivatives, now several reports have verified that HCV can replicate in Huh-7 cells through detection of viral genes as well as viral copy number by Real Time PCR in both cells and supernatant[21]. In the present study, solvent extracts from different plants were tested to determine the antiviral activity against HCV. Real time RT-PCR results showed that *Solanum nigrum *(SN) out of ten medicinal plants showed antiviral effect against HCV. The results demonstrated that methanolic extract of SN showed 37% inhibition a concentration of HCV RNA at non toxic concentration (Figure 2). This extract was further fractionated in different solvents on the basis of polarity. Significant inhibition against HCV was shown by Chloroform extract of *Solanum nigrum *seeds with more than 50% reduction a concentration of viral titer (Figure 3).

#### Inhibition in NS3 region of HCV by *Solanum nigrum *in Huh-7 cells

**Figure 4:**
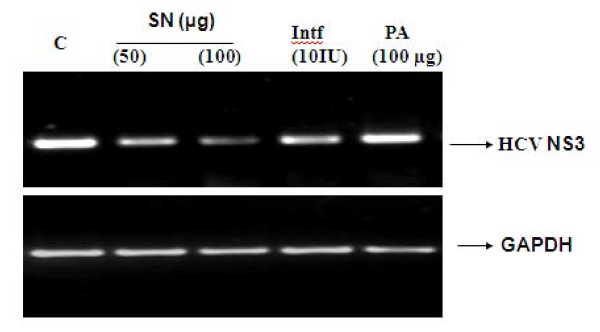
**Antiviral effect of chloroform extract of *Solanum nigrum *against HCV NS3 protease**. Huh-7 cells were transfected with 0.5 μg of constructed HCV NS3 protease vector in the presence and absence of SN and interferon for 24 48 hrs. Cells were harvested and relative RNA determinations were carried out using semi-quantitative RT-PCR. The results demonstrated that SN and interferon inhibit HCV NS3 expression while the expression of GAPDH remained constant.

To determine the effect of S*olanum nigrum *extract against HCV NS3 protease, Huh-7 cells were transfected with NS3 protease plasmid in the presence and absence of herbal extracts. After 48 hrs incubation, cells were harvested, RNA was extracted and cDNA were generated by oligo dT primers. cDNA was amplified by PCR using primers specific to the HCV NS3 protease. Amplification of GAPDH mRNA served as an internal control. Figure 4 demonstrates that *Solanum nigrum *chloroform extract inhibits HCV RNA expression significantly in a dose-dependent manner along with interferon, while GAPDH mRNA expression remains unaffected by the addition of the extract.

## Discussion

HCV infection is a serious global health problem necessitating effective treatment. Currently, there is no vaccine available for prevention of HCV infection due to high degree of strain variation. The current treatment of care, Pegylated interferon α in combination with ribavirin is costly, has significant side effects and fail to cure about half of all infections [22,23]. Hence, there is a need to develop anti-HCV agents from medicinal plants, which are less toxic, more efficacious and cost-effective. Previous studies demonstrated that medicinal plants used for centuries against different diseases including viral diseases and become a focal point to identify, isolate and purify new compounds to treat diseases. Clinical trials have shown that some medicinal herbs might have therapeutic potential for chronic hepatitis C [24]. Many traditional medicinal plants and herbs were reported to have strong antiviral activity like licorice root (*Glycyrrhizia uralensis*). Previous reports showed that extracted substance, glycyrrhizin sulphate, inhibit HIV replication, interfere with virus-to-cell binding and cell-to-cell infection, and induce IFN activity [25]. *Silybum marianum *(milk thistle) has been shown to have clinical applications in the treatment of toxic hepatitis, fatty liver, cirrhosis, ischemic injury, radiation toxicity, and viral hepatitis via its antioxidative, anti-lipid peroxidative, antifibrotic, anti-inflammatory, immunomodulating, and liver regenerating effects [26].

In the present study, methanolic extracts of ten medicinal plants were tested for antiviral activity and *Solanum nigrum *seeds extract was found to exhibit potential antiviral activity against HCV 3a genotype. *Solanum nigrum *(Solanaceae) has been extensively used in traditional medicine in different parts of world to cure tuberculosis, diuresis [27], various nerve disorders [28], ulcer healing [29] liver disorders [30], antiseizure and inflammatory conditions [31]. The methanolic extract of S*olanum nigrum *contains principle components including flavonoids, saponins, alkaloids, phytosterols [32]. According to our finding, methanolic extract of *Solanum nigrum *seeds resulted in 37% reduction in HCV RNA of 3a genotype at non toxic concentration (Figure 2).

Further characterization of antiviral active extracts was performed in different solvents. Our results demonstrated that Chloroform extract of S*olanum nigrum *showed convincing decrease in a concentration of viral titter at non toxic concentration (Figure 3). Previous studies showed that chloroform extracts mainly contains saponins, flavonoids and terpenoids [33-35]. So there could be an excellent therapeutic agent present in this extract that is waiting to identify and characterize through various spectroscopic techniques.

HCV NS3 protease is a target for therapeutic intervention of acute and chronic HCV that NS3 mediated processing of the polyprotein is essential for HCV RNA replication and maturation [36]. Furthermore, NS3 may have other properties involved in interference with host cell functions like inhibition of protein kinase A-mediated signal transduction or cell transformation [37,38]. Previous report demonstrated that ethanol extract from rhizomes of the Chinese medicinal herb *Rhodiola kirilowii (Regel) *exhibited antiviral activity against HCV NS3 serine protease. Twelve compounds were isolated by partitioning of the extract between water and different organic solvents. These compounds were analysed for *in-vitro *antiviral activity against HCV NS3-SP, among which Epicatechin and Epigallocatechin and their dimers has *in-vitro *antiviral activity against HCV NS3-SP [39]. Similarly our data reveals that chloroform extract directed against HCV NS3 resulted in specific inhibition of NS3 protease in a dose-dependent manner while GAPDH remained constant (Figure 4).

On the basis of results presented herein showed that mehanolic and chloroform extract of S*olanum nigrum *seeds play a role in viral clearance during natural HCV infection. These data also suggest that therapeutic induction of extracts might represent an alternative approach for the treatment of chronic HCV infection or the present study leads to the development of more potent and orally available HCV therapeutic drug.

## Abbreviations

**HCV**: Hepatitis C virus; **SN**: *Solanum nigrum*; **PEG-INF**: Pegylated interferon; **SVR**: Sustained Virological Response; **HCC**: Hepatocellular carcinoma; **IRES**: Internal ribosome entry site; **Huh-7**: Human Hepatoma Cell line.

## Competing interests

The authors declare that they have no competing interests.

## Authors' contributions

TJ, UAA and SR contributed equally in lab work and manuscript write up. SDR helped TJ in chemistry techniques. SRD was the principal investigator and provides all facilitates to complete this work. All the authors read and approved the final manuscript.

## Authors' information

Tariq Javed (M.Phil pharmaceutical chemistry), Usman Ali Ashfaq (PhD Molecular Biology), Sana Riaz (M Phil Molecular Biology), Sidra Rehman (MSc Chemistry) and Sheikh Riazuddin (PhD molecular Biology and Dean Post graduate study at Allama Iqbal medical college, Lahore
